# Advancing early relational health: a collaborative exploration of a research agenda

**DOI:** 10.3389/fped.2023.1259022

**Published:** 2023-12-07

**Authors:** Dani Dumitriu, Andréane Lavallée, Jessica L. Riggs, Cynthia A. Frosch, Tyson V. Barker, Debra L. Best, Brenda Blasingame, Jessica Bushar, Dominique Charlot-Swilley, Elizabeth Erickson, Morgan A. Finkel, Bryn Fortune, Leah Gillen, Marty Martinez, Usha Ramachandran, Lee M. Sanders, David W. Willis, Nikki Shearman

**Affiliations:** ^1^ Division of Child and Adolescent Health, Department of Pediatrics, Columbia University Vagelos College of Physicians and Surgeons and NewYork-Presbyterian Morgan Stanley Children’s Hospital, New York, NY, United States; ^2^ Division of Developmental Neuroscience, Department of Psychiatry, Columbia Vagelos College of Physicians and Surgeons, New York, NY, United States; ^3^ Department of Psychiatry, The University of Michigan, Ann Arbor, MI, United States; ^4^ Department of Human Development and Family Science, Auburn University, Auburn, AL, United States; ^5^ Science and Innovation Strategy, Institute for Child Success, Greenville, SC, United States; ^6^ Department of Pediatrics, Division of General Pediatrics and Adolescent Health, Duke University School of Medicine, Durham, NC, United States; ^7^ Vav Amani Consulting LLC, El Cerrito, CA, United States; ^8^ HealthySteps, ZERO TO THREE, Washington, DC, United States; ^9^ Center for Child and Human Development, Georgetown University, Washington, DC, United States; ^10^ Fortune Consulting, Early Relational Health-Family Network Collaborative, Royal Oak, MI, United States; ^11^ Department of Research and Innovation, Reach out and Read, Boston, MA, United States; ^12^ Chief Executive Officer, Reach Out and Read, Boston, MA, United States; ^13^ Department of Pediatrics, Rutgers Robert Wood Johnson Medical School, Piscataway, NJ, United States; ^14^ Department of Pediatrics, Division of General Pediatrics, Stanford University School of Medicine, Stanford, CA, United States; ^15^ Center for the Study of Social Policy, Washington, DC, United States.

**Keywords:** pediatrics, nurture, connection, parents, caregivers, centrality of relationships, early relational health, parent-child interactions

## Abstract

Here, we introduce the Early Relational Health (ERH) Learning Community's bold, large-scale, collaborative, data-driven and practice-informed research agenda focused on furthering our mechanistic understanding of ERH and identifying feasible and effective practices for making ERH promotion a routine and integrated component of pediatric primary care. The ERH Learning Community, formed by a team of parent/caregiver leaders, pediatric care clinicians, researchers, and early childhood development specialists, is a workgroup of Nurture Connection—a hub geared toward promoting ERH, i.e., the positive and nurturing relationship between young children and their parent(s)/caregiver(s), in families and communities nationwide. In response to the current child mental health crisis and the American Academy of Pediatrics (AAP) policy statement promoting ERH, the ERH Learning Community held an in-person meeting at the AAP national headquarters in December 2022 where members collaboratively designed an integrated research agenda to advance ERH. This agenda weaves together community partners, clinicians, and academics, melding the principles of participatory engagement and human-centered design, such as early engagement, co-design, iterative feedback, and cultural humility. Here, we present gaps in the ERH literature that prompted this initiative and the co-design activity that led to this novel and iterative community-focused research agenda, with parents/caregivers at the core, and in close collaboration with pediatric clinicians for real-world promotion of ERH in the pediatric primary care setting.

## Introduction

Prompted by the intersection of widespread recognition of a child mental health crisis and a 2021 American Academy of Pediatrics (AAP) policy statement proposing the promotion of early relational health (ERH) as a strength-based prevention strategy, a team of parent/caregiver leaders, pediatric care clinicians, researchers, and early childhood development specialists was convened in 2022 to bring diverse perspectives to thinking critically and innovatively about best practices and public health policies that advance ERH. This consortium, named the ERH Learning Community, is a workgroup of Nurture Connection (1), a recently launched hub building a national movement to promote ERH defined as the state of emotional well-being that stems from the early positive and mutually nurturing relational bonds between children and their parent(s)/caregiver(s) (1). Invited through an intentional and inclusive process by the instigators of the Learning Community, members were chosen to ensure diverse representation of end users and experts in practice and different research methodologies. The characteristics and contribution of each member are described in Box 1. In December 2022, following five monthly online meetings, the ERH Learning Community gathered for an inaugural 2-day in-person meeting at the AAP national headquarters. No predetermined agenda on specific strategies to be advanced within the ERH realm was set prior to the meeting. Rather, we sought the emergence of consensus through a co-design activity. Here, we summarize the critical problem that brought us together, the solution proposed by the AAP, the gaps in current evidence to actuate this solution, and our proposed co-designed integrated research agenda that emerged from this meeting. We outline a bold, large-scale, collaborative, community-focused, data-driven investigation of the eco-bio-developmental mechanisms (2) associated with ERH, combined with practice-informed research to test real-world feasibility, acceptability, effectiveness, scalability, and impact of light-touch interventions within pediatric primary care aimed at maximizing the power of ERH at supporting wellness and health in families and communities.

Box 1 **ERH Learning Community: Who is Around the Table?**

**Dani Dumitriu, MD, PhD**
  As chair of the COMBO Initiative, Dani brings her dual roles of practicing pediatrician and translational neuroscientist to the design of tools for the observation and promotion of early relational health with a strong emphasis on meaningful real-life outcomes.
**Nikki Shearman, PhD**
  As a leader at Reach Out and Read, Nikki brings perspective and partnership with a network of pediatric primary care clinicians that are seeking to promote the health and well-being of families and their young children through the promotion of positive interactions and connections.
**Tyson Barker, PhD**
  Tyson is a developmental psychologist with extensive experience in measurement and evaluation of early childhood programs. He brings an expertise in rapid-cycle testing and continued quality improvement methods to the network.
**Debra Best, MD, FAAP**
  Debi is a general academic pediatrician who has focused her career on promoting early relational health through clinical practice innovation, pediatric resident education and faculty development, and community systems building efforts.
**Brenda Blasingame**
  Brenda is a social sector consultant with over three decades of experience working on public health issues and early childhood systems development working across issue areas and sectors. She has worked in three sectors: non-profit, government/public and philanthropy at the local, state, and national levels. Her work has focused on systems change efforts that are addressing issues that most impact the lives of children, families and communities marginalized by racial injustices and social inequities.
**Jessica Bushar, MPH**
  Jessica is the research & evaluation director for HealthySteps at ZERO TO THREE, an evidence-based, team-based pediatric primary care program that promotes the health and well-being of babies and toddlers, with an emphasis on positive early relationships. She brings connection to a national network of providers implementing HealthySteps and to a family advisory committee that helps inform the promotion of early relational health as part of the program**.**
**Dominique Charlot-Swilley, PhD**
  Dominique is a clinician, researcher, reflective consultant, and co-developer of Early Relational Health Conversations (ERH-C), with over two decades of working with infants, young children, and families across sectors and communities most impacted by systemic inequities. She served as a regional developer of HealthySteps in the District of Columbia and brings her expertise in infant and early childhood mental health, language equity and lifelong commitment to cultural humility.
**Ellie Erickson, MD, FAAP**
  Ellie is a general academic pediatrician who has focused her career on clinical care and the promotion of early relational health in clinical spaces, focusing on how early literacy activities, including reading with young children, can promote healthy attachment and development. She has served as the medical director for Duke's Reach Out and Read program for the last 8 years and is the inaugural Early Relational Health Fellow for Reach Out and Read.
**Morgan A. Finkel, MD, MS**
  Morgan is a primary care pediatrician and clinician-scientist interested in the potential of early relational health promotion to decrease socioeconomic and racial/ethnic disparities in early childhood cognitive and socioemotional development.
**Bryn Fortune**
  Bryn is a national parent leadership thought leader and coordinator for the ERH-Family Network Collaborative (FNC). Bryn brings the perspective and partnership with 6 parent leaders representing 6 communities and 66 diverse voices core to our Early Relational Health Learning Community effort, including people across the country who identify as Black, African American, and Brown parents; Indigenous parents; parents of children with special health care needs or disabilities; Spanish-speaking immigrant parents; parents with a Southern cultural background; and fathers.
**Cynthia A. Frosch, PhD**
  Cynthia joins as a researcher and endorsed Infant Mental Health Mentor (IMH-E®). She is a firm believer in the power of early relational health to create a more peaceful society and has more than two decades of experience working together with families and practitioners to translate research findings into relevant and actionable steps.
**Leah Gillen**
  Leah joined to offer Reach Out and Read expertise on training.
**Andréane Lavallée, PhD**
  As a postdoctoral fellow, Andréane is interested in studying parent/caregiver-child dyadic emotional connection. She additionally brings her newborn clinical care background as well as training in program development and evaluation.
**Marty Martinez**
  Marty is a public health and non-profit organizational leader who is driving the national office of Reach Out and Read. He brings this experience and advocacy around health equity to drive the critically important impact of early relational health to millions of children and families across the country.
**Usha Ramachandran, MD**
  Usha brings her long-term experience in providing primary pediatric care and Reach Out and Read to diverse families at a federally qualified health center. In addition, she brings her expertise in medical education and experience in leading statewide initiatives to promote early relational health in New Jersey.
**Jessica Riggs, PhD**
  Jessica brings her role as clinical psychologist and researcher, honoring and centering on the perspectives of infant mental health to promote early relationships. She engages in this work as part of Zero to Thrive, an early relational health promotion group at the University of Michigan.
**Lee Sanders, MD, MPH**
  Lee is Professor of Pediatrics and Health Policy at Stanford University, where he directs General Pediatrics, the Health Literacy Lab, and the Complex Primary Care Clinic. With more than 25 years as a Reach Out and Read medical advisor and more than 10 years of interdisciplinary research, he brings early relational health through the lenses of evidence-based practice, health literacy, and human-centered design.
**David Willis, MD**
  David is a national thought leader and champion for advancing early relational health within child health transformation and early childhood. His collaborative leadership at the Center for the Study of Social Policy (CSSP) has resulted in the Nurture Connection movement as a network impact strategy for advancing early relational health.

### Contemporary crises: child mental health and loneliness

The most prominent organizations in children's mental health have declared a national emergency due to the sharp rise in pediatric mental health disorders (3). The critical nature of the situation was first highlighted in 2020 (4) and reiterated in 2022 (5) by the AAP, the American Academy of Child and Adolescent Psychiatry (AACAP) and the Children's Hospital Association (CHA), who joined efforts to tackle the continued rising number of children struggling with mental health disorders across the nation. The February 2022 Center for Disease Control and Prevention Morbidity and Mortality Weekly Report estimated that mental health disorders affect as many as 40% of all children (6). Associated with the child mental health crisis, U.S. Surgeon General Dr. Vivek Murthy officially declared in 2023 an epidemic of loneliness, raising awareness about the long-lasting damaging impacts of social disconnection (7). Human social connection is a complex phenomenon shaped by the structure, functions and quality of relationships (8) stemming from the early-life experiences in the parenting/caregiving environment (9). These national emergencies call for urgent public health research and action to identify and implement effective strategies that strengthen family and community connectedness and promote life-course health and well-being, even in the presence of mental health disorders (10, 11).

### A Major driver of the child mental health crisis: childhood adversity

There is strong evidence that exposure to childhood adversity conveys risk for poor mental and physical health outcomes across the life-course (12–22) and profoundly impacts overall child well-being. The original Adverse Childhood Experiences (ACE) Study assessed seven specific adverse experiences: psychological, physical, or sexual abuse; violence against mother; and living with household members with substance abuse, mental illness/suicidality, or ever imprisoned (23). Addressing a limitation of the original ACE study that was conducted with White, middle-class individuals, further research including more diverse populations expanded the list of ACEs to better represent common experiences (24).

Critically, during discussions following our in-person meeting, the ERH Learning Community recognized post-pandemic trends of spiking poverty and food insecurity (25), parent/caregiver psychopathology (26–35), child maltreatment (36) and neglect (33), breaks in provision of protective services (37), family and domestic violence (38–42), racism (43, 44), parental/caregiver loss (45), social isolation (46) and loneliness (47). Situated in this context, we refer to childhood adversity as an inclusive term that encompasses adverse community environments (e.g., poverty, racism, cyberbullying), and family-level and contextual stressors (48) (e.g., parent/caregiver psychopathology, domestic violence, neglect). This centering of both individual and community experiences to be “representative of America” is an important driver toward our emergent research agenda. We recognize that what works for whom, under what conditions, and toward what benefit, might not be universal. Thus, an inclusive and equitable investigation of the promotion of ERH must be designed to identify the specific needs and, even more importantly, the diverse strengths of different communities.

### A proposed buffer: early relational health

ERH encompasses diverse theoretical constructs including bonding, sensitivity, attachment, and emotional connection, that ultimately converge in their shared objective of describing distinct facets of the parent/caregiver-child relationship. Decades of accumulating evidence points to ERH playing a fundamental role in child physical health, cognitive and socioemotional development, and well-being (49, 50). ERH is also thought to protect against the negative effects of childhood adversity (14, 23, 51–53).

Given the far-reaching implications of strong ERH for child mental health and development (49, 52–54), the AAP published a policy statement in 2021 reorienting pediatric care toward an emphasis on ERH as a promoter of health and well-being across the lifespan (51, 52). Strength-based promotion of healthy early relationships is proposed as a support for families during the current mental health crisis by buffering against childhood adversity, leading to improved health, sense of competence, connection and well-being of both children and their parents/caregivers.

The AAP policy statement also emphasized the importance of developing strategies that focus on early childhood, and the opportunity offered by the pediatric primary care setting for promoting ERH. The first 1,000 days of an infant's life are a critical period for promoting positive and mutually nurturing parent/caregiver-child relationships (11) owing to developmental embedding that has the potential to affect long-term health and well-being. In the face of adversity, a study of 3,500 children showed that the quality of ERH in infancy mattered more than the severity of perinatal adversity in predicting later functioning (55). Pediatric primary care offers standardized, pre-established, consistent, accessible and affordable care in the first few years of a child's life (54–56). It occupies the privileged position of having widespread access to infants, with an estimated well-child visit attendance rate of 63%–93% in the first 3 years of life, even in low income families (57). In commercially insured children, preventative primary care visits increased by 9.9% from 2009 to 2016 (58). Pediatric primary care is delivered in different settings, including within family-centered medical homes where pediatricians are expertly prepared to handle child health needs by providing continuous, affordable, compassionate care with cultural humility (56). Other settings include group well-child care which is thought to increase equitable health care delivery (59) with many documented health benefits for families experiencing marginalization and underserved communities. Benefits include increased adherence to well-child visits, better child nutritional behaviors, increased rates of breastfeeding, optimal child health status and development, and increased parental social support, caregiving behaviors, self-efficacy and psychological well-being (60). Pediatric primary care is thus an ideal setting for widespread implementation of time-efficient and cost-effective strategies focused on promoting ERH (51, 61).

### Identified gaps in promoting ERH: knowledge meets real-world

Prior to our in-person meeting, several of the ERH Learning Community members (AL, MF, DW, NS, DD) contributed to a systematic review and meta-analysis that assessed the global effectiveness of contemporary parent/caregiver-child interventions, initiated within the first six months of life, specifically aimed at improving ERH (62). Consistent with other reviews, we confirmed the effectiveness of the identified interventions in improving a heterogenous set of ERH outcomes including maternal bonding, child attachment, parent/caregiver-child emotional connection, and parent/caregiver emotional availability. However, effect sizes were mostly small-to-moderate and time-limited, with most of the significant effects observed immediately after the intervention ended, and very few studies investigating long-term effects past a few months to years out.

Importantly, our meta-analysis did not provide significant evidence from real-world contexts to support the abundant body of work establishing an association between ERH and later child emotional, mental, and physical health (63–68). These results do not necessarily reflect a lack of causal effect between improved ERH and later child outcomes, but rather underscore the need for additional investigation into the processes and mechanisms underlying the relationship between ERH and life-course health and well-being, as well as exploring the real-world conditions that influence the effectiveness of interventions.

Another identified gap in our systematic review was that only 3 (69–71) of the 93 interventions were implemented by pediatricians or in family-centered pediatric medical homes. Yet it is widely recognized, including in the AAP policy statement, that leveraging pediatric primary care for universal promotion of ERH holds significant potential to yield public health benefits (51).

Additionally, 95% of identified studies focused on biological mothers and only 8% targeted a population at-large rather than focusing on groups with specific risk-factors, pointing to potential lack of generalizability of results to a more inclusive view of families and communities. Finally, we note that no study to-date has included a comprehensive battery of all constructs within the ERH literature (i.e., bonding, attachment, emotional connection, repair, etc.), but rather focus on one or a few of these constructs, limiting the interpretability of which interventions improve which specific ERH constructs. We view these gaps as opportunities that pave the road to a comprehensive research agenda that expands our understanding of underlying mechanisms underpinning the emergence and maintenance of ERH, and uses this knowledge to simultaneously develop and refine effective, equitable, evidence-based ERH interventions. Favorably, our systematic review also found that relational interventions improve ERH outcomes non-dose-dependently, supporting the possibility for scalable light-touch interventions.

## A Co-design activity: emerging vision for a research agenda

To increase the validity of the ERH Learning Community in-person meeting's outcome, no predetermined agenda for the “what” and “how” to usher in best practices in ERH promotion was set prior to our in-person gathering. Rather, the goal was to co-design iteratively and collaboratively an agenda that will lead to an evidence-based framework for effective and equitable promotion of ERH.

Our initial discussion was centered around a vision that advancing ERH can improve life-course health and well-being by improving social cohesiveness and belonging and repair within families and communities. With reference to this vision, we engaged in a co-design process to define a focused problem statement and to identify real-world solutions. Specifically, all members were presented with two sets of prompts, to which they offered independent responses written on sticky notes and placed on four sections of a board. The prompts were: “If we succeed, in 10 years, 1a-What will be the news headline? and 1b-What will families, medical clinicians, and other involved groups post/tweet about it?”; “2a-What do we want to solve (what/where/how/for whom/when)? And 2b-How do we want to solve it?” After responses were posted (*n* = 113), the group discussed convergent and divergent views.

Following the meeting, we applied a human-centered design process (72) to identify prominent themes from the text data (i.e., written responses). Using an inductive approach inspired by thematic analysis, three members of the ERH Learning Community (AL, LG, NS) independently coded the responses for themes and subthemes. Engaging members with firsthand knowledge of the data collection context from the in-person meeting was in line with a human-centered approach, preserving contextual insight, participant perspectives, and the credibility of the outcomes. To increase objectivity, in a confirmation phase, two additional members (JR, CF), one of whom had not attended the in-person meeting and was therefore blinded to the prompts and subsequent conversation (CF), reviewed the data and codes to refine the themes and subthemes to avoid conceptual overlap. Percent agreement between coders in the confirmatory phase ranged from 83.2% to 98.2%. Finally, in a member checking phase (73), themes and subthemes were summarized, presented, reviewed and validated by the ERH Learning Community members. This comprehensive process took place during a follow-up virtual meeting held in February 2023, where 10 members who had attended the in-person meeting provided feedback to ensure the accuracy and credibility of the findings. Given the human-centered design approach, researchers' involvement in the analysis process was deemed fitting to most accurately capture the group's collective vision of the research agenda; however, recognizing the potential for bias, the validation and member checking phase was incorporated to enhance rigor and mitigate subjectivity. In total, 14 subthemes were identified across 3 themes: 1-Goals of the Learning Community agenda; 2-Processes underpinning the agenda; and 3-Strategies to execute the agenda. Examples of direct quotes of written responses for each subtheme that represent the diverse voices in the room are provided in Table 1.

**Table 1:** Examples of written responses.

Subthemes		Written responses
1	Transformation of Care	News headline: *Families and communities leverage power to help shape and inform structural, foundational changes in the healthcare system*.
		Clinician Tweet: *Focusing on ERH has allowed me to not only improve outcomes for my patients but back to the roots of why I went into pediatrics to begin with.*
2	Centrality of Relationship	News headline: *We know our humanity is about our relationships*.
		Tweets by others: *Once you see through a relational lens, you can't unsee it*.
3	Positive Outcomes Across Multiple Levels	Family Tweet: *I feel heard and know I have a trusted partner to help me ensure the health and wellbeing of my child*.
		What do we want to solve: *The lack of relationship-centered strategies driving healthcare delivery for children and families/Pediatric visits/providers to gain understanding of tactics and actions that have impact/yesterday*.
4	Collaborative Learning	How do we want to solve it: *Create learning networks*.
		How do we want to solve it: *Continue to prioritize what families and communities want.*
5	Parents/Caregivers at the Core	News headline: *Families and communities leverage power to help shape and inform structural, foundational changes in the healthcare system*.
		What do we want to solve: *What are simple changes in primary care to improve parent trust? Let's ASK parents!*
6	Values-Based	How do we want to solve it: *Empower parents to redesign well child care from 0 to 5 years*.
		How do we want to solve it: *Improving training, consultation, education, and reflective supervision for providers under guidance of a team protecting against racism and potential harm caused by presentation of data from a biased lens.*
7	Strengths-Based	How do we want to solve it: *Develop new measures of ERH that are strength-based measures*.
		How do we want to solve it: *Create strengths-based and protective boundaries around data use.*
8	Parent/Caregiver-Child-Clinician Partnership	Tweet by policymaker: *Insurance co-pays for parents must be implemented as parents are recognized as co-clinicians*.
		What do we want to solve: *We want to shift the focus of pediatric primary care toward an emphasis of supporting foundational relationships by expanding capacity for this conversation between parents and clinicians during well child care.*
9	Birth to 5 years, with a particular focus on Birth to 3 Years	What do we want to solve: *Revise the well child encounter to support ERH at each visit from 0 to 3*.
		What do we want to solve: *Advocate for a change to the standard appointment time for a well child visit so birth to 3 years is billable to 30 min.*
10	Prioritize Clinician-Parent/Caregiver Encounters	Parent/Caregiver Tweet: *Loved meeting with my doctor today—look forward to trying some new ideas*.
		Clinician Tweet: *Every well child visit is an opportunity for me to connect with loving care and my heart sings each time!*
11	Integrated Within Many Systems of Care	News headline: *Families and communities leverage power to help shape and inform structural, foundational changes in the healthcare system*.
		What do we want to solve: *Support all families in building and sustaining joyful relationships with children starting in pediatric care but eventually in homes and communities.*
12	Using Simple but Impactful Strategies	News headline: *Relationships matter—Keeping it simple transforms health*.
		What do we want to solve: *To find small ways in which pediatricians can inoculate families toward stronger ERH at every well child care visit between 0 and 3 years.*
13	Improved ERH Training	Clinician Tweet: *The best part of my medical school training was how to use the power of my relationships to ensure the health and wellbeing of my patients*.
		What do we want to solve: *In depth assessments of clinician ERH knowledge, beliefs and attitudes; lived experience; capacity.*
14	Iterative, Practice-Informed and Data-Driven Multi-Level Research Methods	How do we want to solve it: *Rapid-cycle testing*.
		What do we want to solve: *How to share the learnings and benefits and how to support adoption of new approaches/peds offices/parents & providers/ongoing in all PDSA processes (Plan, do, study, act iterative cycle).*

ERH, early relational health.

### Theme 1: goals of the learning community agenda

*Transformation of Care* (subtheme 1) was the most prominent subtheme mentioned in 59 written responses. This subtheme translates the Community's goal of transforming and rethinking pediatric primary healthcare. Centering well-child visits on health, wellness, and strengths of the parent/caregiver-child relationship is at the core of this subtheme. *Centrality of Relationships* (subtheme 2) was mentioned in 36 written responses and encompasses the ERH Learning Community's acknowledgement that “relationships matter” and that pediatric care should strive to become relationship-centered. The importance of centering relationships bled into subtheme 3, which focuses the research agenda on *Positive Outcomes Across Multiple Levels* (*n* = 37). Congruent with the ERH literature, a research agenda centered around universal promotion of ERH needs to be outcome-focused, demonstrating improved objective measures of health and well-being. However, the ERH Learning Community also identified the need to measure more subjective outcomes, such as love, joy, empathy, strength, forgiveness, repair, etc., and the importance of measuring outcomes not only in children but also in families and communities. Additionally, adoption of a *Collaborative Learning* approach (subtheme 4; *n* = 9) emerged as a subtheme for participation of pediatric clinicians, parents/caregivers, scientists, and policymakers.

### Theme 2: processes underpinning the agenda

Consistent with our collaborative learning approach, the importance of having *Parents/Caregivers at the Core* (subtheme 5) was present in 37 written responses. Parents/Caregivers partnering at every step with scientists and other collaborators as the research agenda is developed and carried out is critical. As this partnership unfolds, the ERH Learning Collaborative is adopting a *Values-Based* (subtheme 6; *n* = 23), as well as a *Strengths-Based* (subtheme 7; *n* = 5) approach. Principles of equity, diversity, representation, respect, and cultural humility (74) drive not only the relationships among members of the ERH Learning Community but also fundamentally influence how the research agenda will be strategized and implemented. Finally, another emerging subtheme underlying our processes is the focus on the *Parent/Caregiver-Child-Clinician Partnership* (subtheme 8; *n* = 18), building on trust, value, and collaboration to abolish the traditional hierarchical model.

### Theme 3: strategies to execute the agenda

The third theme defined how the ERH Learning Community conceptualized the “who/when/where/how” to be targeted via a comprehensive research agenda. To promote ERH effectively, our work will center on parent/caregiver-child relationships from *Birth to 5 Years, with a Particular Focus on Birth to 3 Years* (subtheme 9; *n* = 13). Efforts will *Prioritize Clinician-Parent/Caregiver Encounters* (subtheme 10; *n* = 21) as opportunities for widespread foundational promotion of ERH, with recognition that our work must ultimately be *Integrated Within Many Systems of Care* (subtheme 11; *n* = 9), eventually extending beyond pediatrics to be inclusive of educational settings, home visiting, and other parent/caregiver-child facing contexts. *Using Simple but Impactful Strategies* (subtheme 12; *n* = 8) is also critical for feasibility and effectiveness. The “how” also included *Improved ERH Training* (subtheme 13; *n* = 8) for pediatric clinicians, and most importantly an *Iterative, Practice-Informed and Data-Driven Multi-Level Research Approach* (subtheme 14; *n* = 27), including new and improved measures of ERH.

The emerging themes define the ERH Learning Community's unified goals, core processes, and strategies, and guide the development of a multi-layered, iterative approach with emphasis on lived experiences and experiential learning leading to innovations.

## Our proposed bold novel research approach

Expanding upon convergent areas of research (11, 12, 51, 52, 75–77), we propose a unique collaboration, inclusive of a broad array of partners with diverse perspectives, with the vision of establishing an evidence base that will drive policy and practice towards hardwiring ERH promotion into pediatric primary care. To rapidly move the needle, we propose a combination of data-driven and practice-informed research methodologies, with parents'/caregivers' and pediatric clinicians' perspectives at the core.

This research approach centers two goals: 1-generate foundational knowledge about ERH in early childhood and its life-course implications, and 2-identify the most feasible and effective practices for making ERH promotion a routine and integrated component of pediatric primary care. To achieve this, we believe it is necessary to connect an extensive network of clinics and clinicians as a platform for field research with a large-scale data-capture effort to acquire longitudinal and cross-sectional parent/caregiver-child ERH and associated outcomes.

### Goal 1: generating foundational knowledge about ERH

Establishing a large prospective ERH-focused cohort of children and their parent(s)/caregiver(s) is critical for deep investigation of the role played by different ERH constructs in health and well-being over the life-course. With increasing understanding for the critical need for “big data”, large multidisciplinary datasets are being established to delve into other key outcomes. Examples include the ABCD study (78) aimed at elucidating the building blocks of brain development from imaging the brains of thousands of children at multiple times during infancy through adolescence, and the RECOVER study (79) aimed at understanding the long-term effects of SARS-CoV-2 infections on a variety of health outcomes. To the best of our knowledge, no ongoing or currently planned similar dataset exists to generate foundational knowledge about ERH.

Goal 1 therefore seeks to expand and leverage existing successful infrastructures (80–83), to develop a large open science dataset by enrolling and following a national cohort of thousands of children and their parent(s)/caregiver(s). Importantly, all efforts will be made to ensure this cohort is “representative of America”, with US census-congruent representation of families of different socioeconomic status, race, and ethnicity.

This data-driven process will ascertain the eco-bio-developmental factors associated with strong ERH and explore the underlying mechanisms of ERH through rigorous data analysis. In collaboration with experts from interconnected and complementary research fields, we will incorporate state-of-the-art methodologies, including machine learning, neural and physiological synchrony, brain imaging, genetics, medicine, and developmental science to establish the foundations of ERH. Importantly, this exploration of ERH will not focus on singular constructs within the field, such as bonding, attachment, emotional connection, or emotional availability, but rather seek to collaboratively generate the most comprehensive picture of ERH through all available lenses and in the context of every family's unique experience, structure, and strengths. For rapid generation and dissemination of actionable knowledge, this dataset will be freely and openly shared with the entire ERH community, with anyone joining the overarching Nurture Connection hub.

A successful example of this type of data-driven process is currently employed by the Center on the Developing Child at Harvard University, which has spearheaded the dissemination of concepts such as toxic stress (84) and serve and return (85), as well as implementation of evidence-based practices building on these concepts (86). Strengths of utilizing this process include the ability to conduct rigorous analyses to isolate mechanisms of change that emphasize internal validity (87, 88), and a large prospective design to identify predictors of ERH (89). In alignment with the ERH Learning Community's research agenda discussion resulting from the co-design activity, this data-driven process will innovate by emphasizing a strengths-based focus on positive predictors, mediators, and outcomes of ERH, including positive childhood experiences (PCEs) as opposed to ACEs, and flourishing and thriving as opposed to mental health disorders.

### Goal 2: ERH promotion in pediatric primary care

The practice-informed process brings the research within the context of practice (90) to explore together with families, practitioners and communities what strategies work, for whom, and under which conditions. Consistent with the themes in our co-design activity, we propose to use the established Reach Out and Read large network of pediatric primary care clinics and clinicians as a platform for the practice-informed process. The Reach Out and Read model has been integrated successfully into more than 6,000 clinics across the U.S. and is an ideal platform for practice-based research on ERH. There is a strong evidence base documenting the effectiveness of integration of Reach Out and Read into pediatric primary care (91). Critically, this network offers broad representation from a range of pediatric primary care settings, including all major US geographic areas, rural and urban settings, and professional contexts (e.g., academic/private/community practices and pediatric/family medicine specialties). Drawing from broader literature, initiatives embedded in primary care, such as the Developmental Understanding and Legal Collaboration for Everyone (DULCE) approach, have demonstrated the advantages of leveraging well-child visits to improve pediatric health care and family outcomes at scale (92). As noted previously, limited experimental evidence is available to support timely implementation of evidence-based interventions in the realm of ERH (62). Given the uniqueness of this endeavor, though, we propose starting with pediatric primary care as a foundation, in partnership with Rearch Out and Read, which may lead to rethinking primary care (61) and to new avenues for further exploration.

We will employ Community Activated Research [i.e., engaging parents/caregivers/communities in the research process and giving their voices high priority (93)] to ensure integration of co-design with parents/caregivers, clinicians and communities. To generate knowledge within practice, community activated research will be supplemented by methodologies such as real-world pragmatic designs (94), qualitative research, continued quality improvement [CQI; e.g., quick learning cycles, such as Plan-Do-Study-Act (PDSA)], implementation science (95). Knowledge generated can be used for internal improvement within an organization (96), or disseminated through learning networks (97).

Our research will include determination of the factors that strengthen the clinician-parent/caregiver-infant relationship and ensure integration of effective real-world promotion of ERH into pediatric primary care; development and evaluation of tools to equip pediatric clinicians to both observe and promote ERH; and cultivation of adaptations of the well-child visit that provide opportunities for interventions that effectively advance ERH.

This practice-informed process, which emphasizes external validity, will support the identification of ERH innovations that can be successfully implemented within a variety of health care and related settings (e.g., team-based care, family practice), and with diverse communities. Current successful examples of this methodology include the Home Visiting Collaborative Improvement and Innovation Network (HV CoIIN), which employs CQI to improve the quality of home visiting programs (98), and the IDEAS Impact Framework, which employs rapid-cycle testing to generate evidence for early childhood programs (99). Strengths of this process include higher likelihood of successful implementation due to stronger understanding of practical considerations, shorter timeline to share and scale evidence-informed innovations, and greater considerations of cultural aspects that may influence ERH and its promotion within the pediatric primary care setting.

### The homerun: combining evidence-based practice and practice-informed evidence

The most innovative aspect of our research approach is our intention to utilize both evidence-based practice and practice-informed evidence methodologies in a seamless and integrated manner. In other words, to operationalize this approach, we will leverage the large dataset established in our data-driven process to explore mechanisms that support ERH (and the individual variation in the influence of eco-bio-developmental factors on ERH) and feed this knowledge into our practice-informed research. At the same time, the practice-informed research will define feasible practice to cycle back and further test through the data-driven process. The evidence-based practice and practice-informed evidence research will be operated simultaneously, in continuous cycles, each with their specific research methodologies. Findings will be disseminated through our network to inform future cycles of evidence-based practice and practice-informed evidence research. For example, research questions can be generated by any member or affiliate of the ERH Learning Community, including academic researchers, parents/caregivers, and clinicians. Our extensive dataset will allow for a rapid examination of both predictors of ERH, as well as how ERH predicts later outcomes. Identified mechanisms will inform the development of promising new practices that improve ERH. Likewise, promising strategies arising from the practice-informed methodology will inform new hypotheses for mechanisms underlying strong ERH. This combined approach is uniquely promising in answering: *what works* for improving ERH, *for whom* do certain supports/interventions work, and *under what contexts* do these supports/interventions work best (100, 101). In conjunction with traditional hypothesis-driven research (102), we will leverage increasingly popular inductive analytic processes to explore patterns both within the large datasets, as well as through practice-based innovations (103). This inductive, dual-research approach will allow for a high level of collaboration and connection between both research methodologies, ensuring both internal and external validity are valued and maximized throughout the learning and innovation process (104).

The culmination of this integrative research approach will be to achieve population health impacts. We anticipate our preliminary work in the prior years will have demonstrated both testable and promising strategies that improve ERH and subsequent child development, as well as the conditions and context necessary to embed and scale a program or set of practices. Once these advances are achieved, it will be critical to test our assumptions and to demonstrate the impact of our intervention program to generate the level of evidence required for widespread policy changes. We anticipate this demonstration will result from large-scale rigorous mixed methodologies, including a randomized controlled trial (RCT) as a strong basis for supporting causal inference between our proposed innovations and population health impacts. Findings will be communicated through traditional scientific dissemination of results through publications and presentations as well as to a variety of audiences through Nurture Connection (e.g., policymakers, early educators, health care clinicians) to broadly support dissemination and implementation of identified evidence-based practices that support ERH.

## ERH learning community's commitment to continued self-reflective practice

Given the explicit commitment of the ERH Learning Community to creating an inclusive space for ERH conversations to occur with *parents/caregivers at the core* of a *values-based* approach, we are committed to continued examination of our biases. In an online meeting of the ERH Learning Community to review a draft of this article, discussion reflected on the importance of ensuring that promotion of ERH occurs in partnership with parents/caregivers; that we appreciate the systemic barriers to health and well-being faced by many under-resourced and marginalized communities; that we embrace the wide range of adults that participate in raising children across a diversity of cultures; that we recognize the negative connotation for many families of words like “resilience” and “stakeholder.” This discussion prompted attention to these considerations throughout this article, in particular in our Reflexivity Statement (Box 2).

Box 2 **Reflectivity Statement.**

At the heart of the Early Relational Health (ERH) Learning Community's research agenda is a commitment to adopting intersectional-based principles and integrating values of equity, diversity, and inclusion. We are dedicated to sustaining a research environment that represents the wide range of lived experiences including those of marginalized and underrepresented groups.

As a note on terminology, we appreciate and recognize that children are cared for by any number of adults (e.g., parents, grandparents, extended family, foster parents). To identify the most inclusive term, the ERH Learning Community consulted parent leaders in the ERH Family Network Community, a separate workgroup within Nurture Connection led by one member of the ERH Learning Community (B.F.). These parent leaders represent people across the country who identify as Black, African American, and Brown parents; Indigenous parents; parents of children with special health care needs or disabilities; Spanish-speaking immigrant parents; parents with a Southern cultural background; and fathers. Based on their recommendation, here, we use the term “parent/caregiver” as an umbrella term inclusive of any adult responsible for the consistent care and well-being of the child.

Going forward, to supplement the process of co-design activity presented here, a survey expanding on the 3 themes and 14 subthemes will be developed to explore the wider parent/caregiver and clinician communities' perspective on promotion of ERH. Such continued self-reflection may result in revisions and adjustments of the ERH Learning Community's work.

## Conclusion

In response to the 2021 AAP policy statement calling for promotion of ERH as a buffer of childhood adversity, the ERH Learning Community co-designed a research agenda to establish an evidence-base that will drive policy and practice within pediatric care to stimulate life-course health and well-being through universal promotion of ERH. We believe that our opportunity for success lies in bringing together a wide range of expertise in this work, including parents, practitioners, and researchers and by leveraging the unique strengths of both a successful academic research longitudinal cohort of parents and young children and an established network of pediatric primary care practices. Our research proposal encompasses a participatory approach with parents/caregivers at the core, that iteratively combines data-driven and practice-informed methodologies to generate foundational knowledge about the eco-bio-developmental factors associated with strong ERH and develop strategies for real-world promotion of ERH in the pediatric primary care setting.
